# The Never Given 2022 Pittendrigh/Aschoff Lecture: The Clock Network in the Brain—Insights From Insects

**DOI:** 10.1177/07487304241290861

**Published:** 2024-11-11

**Authors:** Charlotte Helfrich-Förster

**Affiliations:** Neurobiology and Genetics, Theodor-Boveri-Institute, Biocenter, University of Würzburg, Würzburg, Germany

**Keywords:** activity rhythm, period, pigment-dispersing factor, cryptochrome, rhodopsins, 2-oscillator model

## Abstract

My journey into chronobiology began in 1977 with lectures and internships with Wolfgang Engelmann and Hans Erkert at the University of Tübingen in Germany. At that time, the only known animal clock gene was *Period*, and the location and organization of the master circadian clock in the brain was completely unknown for the model insect *Drosophila melanogaster*. I was thus privileged to witness and participate in the research that led us from discovering the first clock gene to identifying the clock network in the fly brain and the putative pathways linking it to behavior and physiology. This article highlights my role in these developments and also shows how the successful use of *D. melanogaster* for studies of circadian rhythms has contributed to the understanding of clock networks in other animals. I also report on my experiences in the German scientific system and hope that my story will be of interest to some of you.

First, I would like to thank Orie Shafer for inviting me to give the Pittendrigh-Aschoff talk at the 2022 SRBR meeting in Amelia Island. This was a great honor and I really wanted to talk about my scientific journey in chronobiology. But things turned out differently, and I couldn’t travel because my partner had a serious accident 3 days before the conference started. Fortunately, now, 2 years later, he is well again, and I have decided to finally write down what I originally wanted to say. I also couldn’t resist adding scientific work that took place after 2022.

My scientific career is in several aspects unusual. First, I worked throughout my career on the same self-selected subject. Second, I was for many years without payment (my doctoral work and large parts of my post-doctoral time were unpaid meaning that I was working half-a-day as a scientific drawer to earn my livings), and third, I paused to raise my 2 children and became a permanent professor only when I was already 52. I thought that these personal things might be interesting for the younger generation. Therefore, I decided to write not only about science but also about my scientific motivation and personal shortcuts. Many thanks go to my scientific mentors, supporters and collaborators: Wolfgang Engelmann, Erwin Bünning, Karl-Friedrich Fischbach, Bronislaw Cymborowksi, Jeffrey C. (Jeff) Hall, Martin Heisenberg, Hans Erkert, Uwe Homberg, Monika Stengl, Ralf Stanewsky, Stephan Schneuwly, Alois Hofbauer, John Ewer, Rodolfo Costa, Paul Taghert, Orie Shafer, Guy Bloch, Fernanda Ceriani, David Martinez-Torres and many others. I am similar thankful to all undergraduate and PhD students as well as postdoctoral students, who decided to work with me. Here, I will only name a few: Dirk Rieger, Taishi Yoshii, Pamela Menegazzi, Matthias Schlichting, Pingkalai Senthilan, Katharina Beer, Peter Deppisch, Giulia Manoli, and Nils Reinhard.

I am a chronobiologist with heart and soul. My love for chronobiology already began in the second semester of my biology studies, shortly after I moved from the University of Stuttgart to the University of Tübingen in 1977. At that time, I took a seminar with Hans Erkert on the suprachiasmatic nuclei (SCN) and their function in mammals and was fascinated by how these small nuclei can control the circadian behavior of the whole animal. I was about to delve deeply into SCN matters, when I discovered that there was a second person at the University of Tübingen who gave lectures about biological rhythms: Wolfgang Engelmann. In his lectures, Wolfgang covered the entire chronobiology from ultradian, tidal, circadian, semilunar, lunar and annual rhythms and he talked about such rhythms in unicellulars, fungi, plants, animals and humans. Every semester, he taught a different chronobiological topic, and I started to listen to all his lectures. During several practical courses, I became interested in the circadian rhythmic leaf movements of *Phaseolus coccinus* and *Oxalis regnellii*, the ultradian leaflet movements of *Desmodium gyrans*, the petal movements of the Flaming Katy, *Kalanchoe blossfeldiana*, the transpiration rhythms of oat (*Avena sativa*), and the rhythmic formation of conidia in the bread mold, *Neurospora crassa*. Furthermore, I learnt how to record the locomotor activity of golden hamsters, squirrel monkeys, cockroaches and flies, and I participated in excursions to the University of Cologne for monitoring lunar rhythms in the marine midge *Clunio marinus* with Dietrich Neumann and to Erling-Andechs to visit Jürgen Aschoff’s famous bunker and to talk about the there-ongoing human experiments.

I also participated in self-experiments on human rhythms. One of Wolfgang’s ideas at that time was that the maximal body temperature that could be reached after excessive physical exercise varies throughout the 24-h day and that caffeine could influence this rhythm. The only way to find out was to measure core body temperature while cycling on an ergometer at different times of the day, once without and once with caffeine. I forgot the outcome of this experiment, but I still remember that it took me at least 40 minutes of hard exercise until my body temperature was reaching the maximum, that this experiment was especially hard at 3am, and that caffeine did neither help nor effect the outcome. Other more relevant self-experiments looked at the effects of lithium chloride on the free period of humans. Lithium had been used to treat bipolar disorders and major depression in human patients for decades and it appeared to slow the circadian clocks of most plants and animals (Engelmann, 1972; Hofmann et al., 1978). Since certain rhythmic parameters in some human patients had short periods that became desynchronized from the body temperature rhythm during depression (Pflug et al., 1982), Wolfgang wanted to test the hypothesis that lithium has a beneficial effect on depression by lengthening the period of short-period rhythms and normalizing the phase relationship of all rhythms. Together with his long-time collaboration partner Anders Johnsson (biophysicist at the Norwegian University of Science and Technology, Trondheim) and Burkhard Pflug (1939-2009; psychiatrist at Frankfurt University Hospital), he planned to test the effect of lithium on healthy human volunteers during the Arctic summer in Spitsbergen under free-running conditions. I wanted to take part in this study as a volunteer and only backed out because my partner at the time was not at all enthusiastic about the idea. The adventurous experiment took place in 1978 (see Engelmann, 2010, for a detailed report), and it turned out that lithium slows down the speed of the human body clock (Johnsson et al., 1983).

What I like most about chronobiology is its interdisciplinary nature: one needs some knowledge of physics and mathematics to understand oscillations, knowledge of biochemistry to know what is going on in cells, and knowledge of many biological disciplines to understand the clocks in different organisms. In addition, chronobiology clearly has medical implications for humans and, as I now know, for ecosystems as well. In addition, circadian clocks are important for measuring the length of the day and preparing organisms in advance for the coming seasons—a theory first put forward by Erwin Bünning, who was still working at the Tübingen Botanical Institute at the time (see Helfrich-Förster, 2024a for Bünning’s and Engelmann’s contribution to photoperiodism).

In 1981, I decided to perform the practical work for my diploma thesis in chronobiology and asked Wolfgang for a thesis topic. He suggested several topics on plants, but also one on flies: the use of different brain mutants in *Drosophila melanogaster* to find the location of the master clock in the brain. I opted for the latter, without realizing that this topic would keep me busy to this day.

## On the Search of the Master Clock in the Brain of Fruit Flies with the Help of Neural Mutants

**Figure 1.**
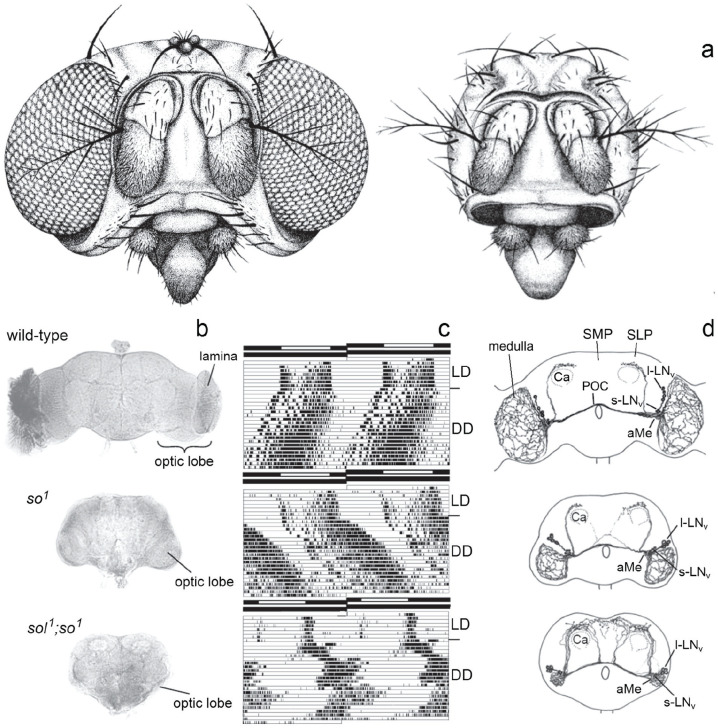
Deciphering the circadian clock of *Drosophila melanogaster* using eyeless neural mutants. (a) Drawings of a wild-type and *sine oculis* (*so*^*1*^) head. (b) Brain morphology of the wild-type and the eyeless mutants *so*^*1*^ and *sol*^*1*^; *so*^*1*^. Due to the missing input of the photoreceptor cells and the consequent death of optic lobe interneurons, the eyeless mutants lack the lamina and the rest of the optic lobe is considerable smaller than in wild-type flies. The optic lobe of *so*^*1*^ mutants has about 40% and that of *sol*^*1*^; *so*^*1*^ about 5% of the wild-type size. (c) Double-plotted actograms revealing locomotor activity rhythms of individual flies under light-dark (LD) cycles and constant darkness (DD). Despite the strongly affected brains, both mutants entrain to the LD cycles and show rhythmic, circadian activity patterns under DD, although with different appearance. The free-running period of the *so*^*1*^ mutant is significantly longer than that of the wild-type fly; the *sol*^*1*^; *so*^*1*^ mutant shows simultaneously 2 free-running components. The black-white bars on the top of each actogram represent the LD schedule. (d) Arborization pattern of neurons stained with an antibody against the crustacean pigment-dispersing hormone (PDH), a homolog of the insect pigment-dispersing factor (PDF). There are 8 PDF neurons per brain hemisphere, the 4 with large somata are called “large ventrolateral neurons” (l-LN_v_) and the 4 with small somata “small ventrolateral neurons” (s-LN_v_). Both types of PDF neurons arborize in the accessory medulla (aMe). The s-LN_v_ project into the superior lateral protocerebrum (SLP), terminating close to the calyces (Ca) of the mushroom bodies. The l-LN_v_ project into the medulla and via the posterior optic commissure (POC) to the other hemisphere. In *sol*^*1*^; *so*^*1*^ mutants, the optic lobe fibers run to the SLP and superior medial protocerebrum (SMP) instead of projecting in the tiny medulla. Modified after Helfrich-Förster (2014).

At that time, the first clock mutants in the clock gene *period* (*per*^*s*^, *per*^*l*^, and *per*^*0*^) had been identified (Konopka and Benzer, 1971). Transplantation experiments had indicated that the circadian clock is located in the brain and humoral factors are involved in transferring circadian signals to the locomotor centers in *Drosophila* (Handler and Konopka, 1979); however, the site of the clock in the brain of flies was completely unknown. Only in larger insects such as moths the clock had been successfully traced to the superior central brain (Truman, 1972, 1974) and in cockroaches, beetles and crickets to the optic lobes (Koehler and Fleissner, 1978; Page, 1982; Wiedenmann, 1983). To test whether the optic lobes do also house the fly’s circadian clock, my task was to investigate the rhythmic behavior of neural mutants with small optic lobes. A previous PhD thesis suggested that some individuals of eyeless *sine oculis* (*so*^*1*^) mutants (Figure 1a) had lost their rhythmic activity pattern under constant darkness (DD) (Mack and Engelmann, 1981). Since it was not clear whether these arrhythmic animals lacked a part of the optic lobe that the rhythmic ones still possessed, I first had to record the locomotor activity of the individual flies and then to analyze the structure of their brains by histological means. To my great disappointment, I found that the optic lobes had the same small size in all eyeless mutants (Figure 1b) and that virtually all mutant flies showed rhythmic behavior under DD (Helfrich and Engelmann, 1983), just their free-running period was longer than that of wild-type flies (Figure 1c). Wolfgang suggested that I change my PhD thesis research focus to *Drosophila* sleep in my PhD thesis and took me to my first scientific meeting in Zürich, a meeting on sleep research, where I met Irene Tobler and Alexander Borbely. Irene impressed me by her sleep studies in cockroaches and scorpions (Tobler, 1983; Tobler and Stalder, 1988), but I remained reluctant to study sleep in *Drosophila.* First, I wanted to locate the circadian clock in the brain of the fly. Wolfgang took me also to the Gordon Research Conference in Colby-Sawyers College in 1983, where I could present the results of my diploma thesis and had the opportunity to meet many colleagues in the circadian field. Woody Hastings even invited me to give a seminar in his group at Harvard University after the GRC, and he taught me how to record luminescence rhythms (glowing and flashing) in *Gonyaulax polyedra* (now *Lingulodinium polyedra*). All this was very exciting for me.

**Figure 2.**
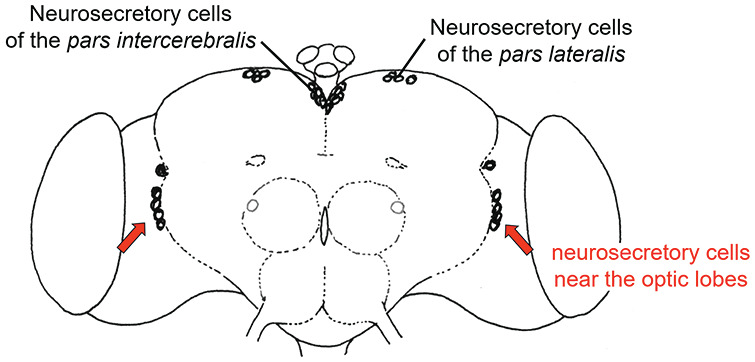
Neurosecretory cells in the brain of wild-type *D. melanogaster* stained with paraldehyde fuchsin.

Back in Germany, I continued the search for the master clock in flies in my doctoral studies. I cooperated with Karl-Friedrich Fischbach (University of Freiburg) who had just generated double mutants of *so*^*1*^ and *small optic lobes (sol*^*1*^), which he gave me prior to publication (Fischbach and Technau, 1984). The *sol*^*1*^; *so*^*1*^ mutants lacked all optic lobe neurons except large medulla tangential neurons, and as a consequence, they had extremely tiny optic lobes (∼5% of wildtype size) (Figure 1b). To my surprise, the *sol*^*1*^; *so*^*1*^ mutants had a very different activity pattern than the previously investigated mutants (Figure 1c): As soon as they were transferred to DD, these showed 2 simultaneously free-running activity components, one with a long and the other with a short period. I concluded that the optic lobes are important for maintaining a coherent activity pattern and speculated that they might be involved in the coupling of independent circadian oscillators (Helfrich, 1986). The large medulla tangential neurons were good candidates for circadian pacemaker neurons and I assumed that their shape might be altered in *sol*^*1*^; *so*^*1*^ mutants; but at this time I had no tool to label them individually. Three years later, Dushay et al. (1989) found with *disconnected* (*disco*^*1*^) the first arrhythmic optic lobe mutant. In this mutant, virtually all optic lobe neurons died during development, undermining the putative role of the medulla tangentials as circadian pacemaker neurons. Subsequently, I tried to identify these medulla tangential neurons that were still present in *sol*^*1*^; *so*^*1*^ mutants, but obviously absent in *disco*^*1*^ mutants. Since Handler and Konopka (1979) could reinstall short-period rhythms in arrhythmic *per*^*0*^ flies by transplanting *per*^*s*^ brains into the abdomen of *per*^*0*^ mutants, I was especially interested in identifying neurosecretory cells that may release their neuropeptides into the hemolymph of the flies. To do so, I went to the lab of Bronislaw Cymborowski (University of Warsaw) where neurosecretory cells in the brains of insects were routinely stained with paraldehyde fuchsin. Indeed, we found 4 to 5 large neurosecretory cells at the lateral border of the central brain and the optic lobes in wild-type flies and *sol*^*1*^; *so*^*1*^ mutants that were absent in *disco*^*1*^ mutants, which underlines the putative importance of neurosecretory medulla tangentials for circadian rhythmicity. However, this work was never published. In addition, we surgically removed the optic lobes of *Musca domestica* to confirm their importance for rhythmic behavior in larger flies. We found that the activity rhythm disappeared only when the lateral brain was additionally lesioned (Helfrich et al., 1985). Since the neurosecretory medulla tangentials have their cell bodies at the boundary between the lateral brain and the optic lobes (Figure 2), this finding supports the possibility that they are the circadian pacemaker neurons in question in larger flies as well.

I finished my thesis in 1985 with the best possible grade and was awarded the “Attempto-Prize” of the University Tübingen, which came with money for scientific traveling. Immediately after the defense, my son Christian was born, which prevented me from doing a planned postdoctoral stay in the USA, as my husband could not simply move and accompany me. Nevertheless, the money from the Attempto-Prize enabled me to frequently attend scientific conferences where I presented my results.

## The first specific labeling of clock neurons with an antibody against the pigment-dispersing factor (pdf)

**Figure 3.**
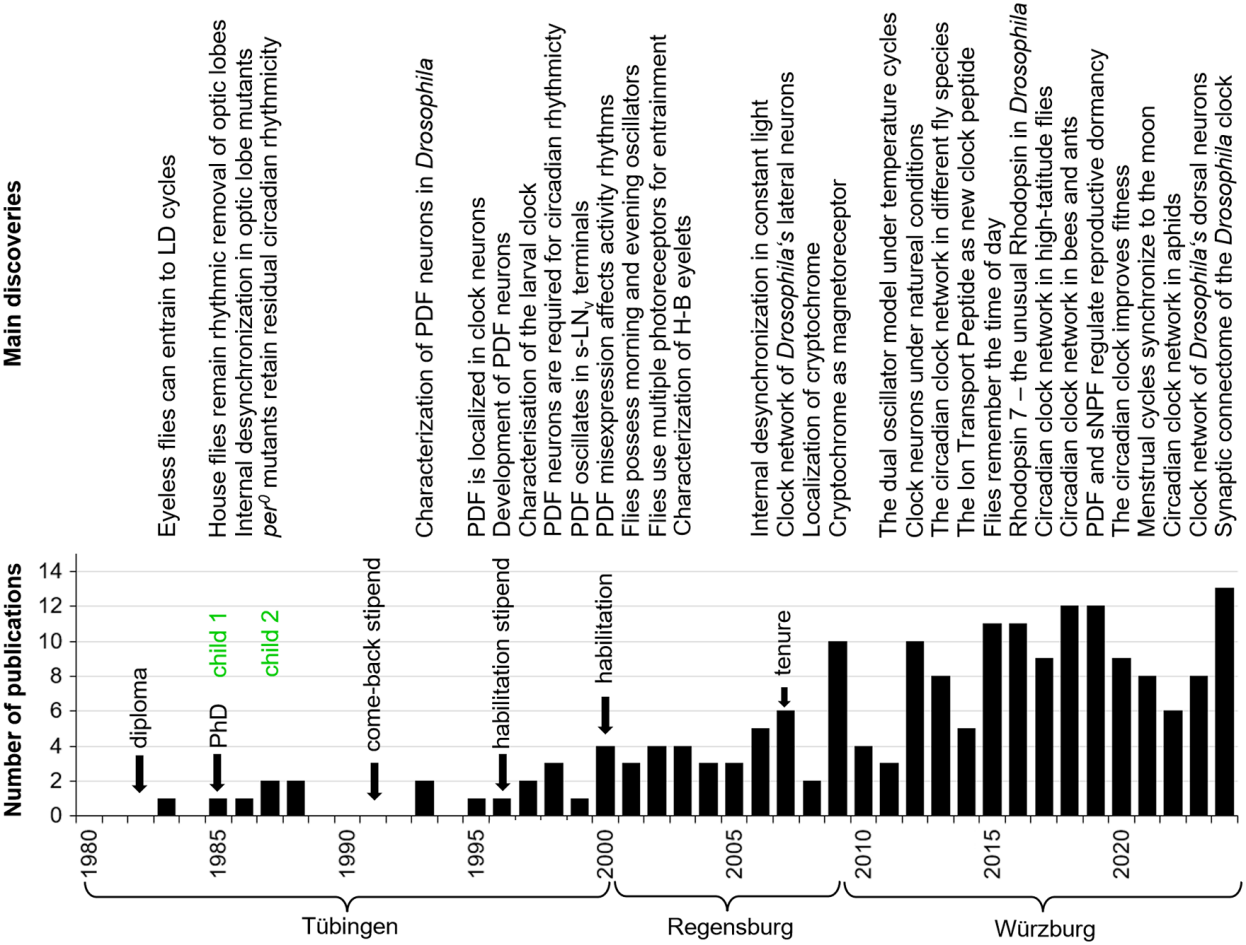
Timeline of my most important scientific discoveries, personal life events and number of publications. The lack of publications after the birth of my 2 children and the resulting delay in career development is clearly visible. It was only after I received a position at the University of Regensburg and, in particular, after I was tenured that the number of publications rose steadily.

Since the paraldehyde fuchsin staining labeled only the cell bodies of the neurons, I was looking for an antibody that specifically labels the whole neurites of the putative tangential medulla cells that might be involved in the master circadian clock of flies. After the birth of my daughter Mareike in 1987 and a period of maternity leave, I was given the opportunity to carry out these studies in 1991 with the help of a 1-year scholarship from the University of Tübingen (a kind of “come-back” stipend; see Figure 3). At the Gordon Research Conference on Chronobiology in Irsee in 1992, I met Monika Stengl who presented a poster showing the immunostaining of cockroach and cricket brains with an antibody against the crustacean Pigment-Dispersing Hormone (ß-PDH) which also stains Pigment-Dispersing Factor (PDF) positive neurons of insects (Homberg et al., 1991). ß-PDH and PDF are neuropeptides that are used as neuromessengers in interneurons of panarthropoda (Mayer et al., 2015). In crustaceans, ß-PDH is responsible for the daily distal pigment dispersion in the compound eye, which protects the photoreceptor cells from too much light (Rao and Riehm, 1993). This rhythmic pigment migration continues even under constant darkness indicating that it is controlled by the circadian clock. As neuromessengers, ß-PDH and PDF are present in the neurites, and the antibodies labeled the arborizations of the entire neurons. It was immediately clear to me that these neurons are the ones I was looking for, and in cooperation with Uwe Homberg, we immunostained wild-type and mutant *Drosophila* brains with anti-ß-PDH (Helfrich-Förster and Homberg, 1993). *Drosophila* anti-PDF gave a virtually identical staining pattern as revealed later by Jae Park in the group of Jeff Hall (Park et al., 2000), and I will therefore simply call the stained neurons “PDF neurons.” We found 8 PDF neurons (4 with small and 4 with large somata) per brain hemisphere, which were located between the central brain and the optic lobes (Helfrich-Förster and Homberg, 1993; Figure 1d). The 4 large PDF neurons were indeed medulla tangential neurons, while the 4 small PDF neurons were not, but projected into the superior lateral protocerebrum and terminated dorso-anteriorly of the calyces of the mushroom bodies, centers for learning and memory (Figure 1d, top). All 8 PDF neurons were absent in *disco*^*1*^ mutants (Helfrich-Förster and Homberg, 1993).

The next logical step was to test whether the PDF neurons belong indeed to *Drosophila’s* circadian clock neurons. Fortunately, in the meantime, Kathy Siwicki in Jeff Hall’s lab had generated an antibody against PER that revealed circadian clock neurons in the lateral and dorsal brain of the fly that were accordingly called lateral and dorsal neurons (LN and DN) (Siwicki et al., 1988; Zerr et al., 1990; Ewer et al., 1992). A co-labeling of PER and PDF positive neurons showed that the PDF neurons were identical with 2 subgroups of the LN—the large and small ventral lateral neurons (l-LN_v_ and s-LN_v_) (Helfrich-Förster, 1995). Thus, the PDF neurons were a subgroup of the circadian clock neurons.

As typical for medulla tangential neurons, the large PDF neurons (l-LN_v_) of wild-type flies form a wide fiber network in the distal medulla (Figure 1d). In addition, they send collaterals via the posterior optic commissure into the contralateral medulla. In *so*^*1*^ mutants, the overall arborization pattern of the l-LN_v_ appears largely normal; except for the fact that it is denser in the medulla and that sometimes few fibers of the l-LN_v_ project into the superior lateral and medial protocerebrum. The latter seem to follow the projections of the small PDF cells (s-LN_v_). Such misrouted l-LN_v_ fibers can occasionally be observed also in wild-type flies, but much more frequently in flies with small optic lobes (Helfrich-Förster and Homberg, 1993). In flies with miniature optic lobes such as *sol*^*1*^; *so*^*1*^ double mutants, all l-LN_v_ seemed to run toward the superior protocerebrum instead of into the distal medulla (Figure 1d), most probably because their original targets in the distal medulla had disappeared. Although it is strongly suggestive that the altered morphology of the l-LN_v_ is the cause for the appearance of the 2 free-running components in behavioral rhythmicity under DD, it took another decade to prove (see below).

I was quite satisfied with my results and wanted to continue the research on the PDF neurons and the origin of the 2 free-running activity rhythms in the mutants with tiny optic lobes, but my fellowship could not be prolonged, and my efforts to get a grant for my habilitation (the German requirement for working as a professor) were not successful. So, it was extremely doubtful whether I could continue at all. Therefore, in 1994, I took for some time a job as a research assistant at the Max-Planck Institute (MPI) of Biological Cybernetics and worked on the effects of anesthetics in the guinea pig brain (Antkowiak and Helfrich-Förster, 1998). The time at the MPI was quite interesting because I learnt many new things and because I met Steven de Belle who worked on the *Drosophila* mushroom bodies (MBs) and their role in learning and memory. I immediately had 2 ideas: (1) to test whether the MBs are on the output pathway to locomotor activity rhythms, (2) to test whether the PDF terminals of the s-LN_v_, which terminated close to the calyces of the MBs signal to them and affect learning in a circadian manner. We decided to ablate the MBs and first analyze the effects of this ablation on locomotor activity rhythms. We found that the MBs are not on the output pathway of the clock neurons toward the control of locomotor activity. However, we detected that the flies without MBs were significantly more active than the wild-type controls meaning that that the MBs are important for resting behavior of the flies, a result that we published 8 years later (Helfrich-Förster et al., 2002). We did not dare to call this resting behavior sleep, but in 2006, Amita Sehgal and coworkers published a paper in Nature with the title “Sleep in *Drosophila* is regulated by adult mushroom bodies” (Joiner et al., 2006). In the meantime, we know that that there are several sleep centers in the fly brain (including the MBs) and that these get circadian input from the circadian clock neurons (e.g., Helfrich-Förster, 2018; Bertolini and Helfrich-Förster, 2023). Thus, we have been on the right track already in 1994. Many years later, in collaboration with Martin Heisenberg, we found that the circadian clock and its signals to the MBs enable flies to memorize the time of day (Chouhan et al., 2015).

In 1994, before my PNAS paper was published (Helfrich-Förster, 1995), Jeff Hall learnt that I could successfully label some clock neurons with anti-PDH and invited me to Brandeis. From that time on, we started a fruitful collaboration. Together with Martin Heisenberg, Jeff also intervened with the German Research Foundation and informed them that the project I had applied for was rejected because of unfair reviewer statements. Indeed, I was allowed to apply for a smaller 2-year grant on the same topic, which I got and started in 1996. Afterwards, I successfully applied for a habilitation position funded by the Margarete-Wrangell Program of the country Baden-Württemberg (Germany). This enabled me to gain my *venia legendi* (the confirmation of teaching qualification for my habilitation) in zoology in December 2000 at the Zoological Institute of the University of Tübingen. During my work for the habilitation, I was hosted by the laboratory of Hans Erkert. Again, this was an interesting time, because I was able to experience at firsthand how Hans Erkert carried out his experiments with nocturnal and diurnal monkeys and how he discovered that the activity of owl monkeys (*Aotus azarai*) in the wild was strongly influenced by moonlight (Fernández-Duque et al., 2010). These studies sparked my interest in direct light effects on the circadian clock (also called masking), which later led me to investigate the effects of moonlight on flies (Bachleitner et al., 2007; Kempinger et al., 2009; Schlichting et al., 2014, 2015a). In addition, they founded my interest in lunar rhythms (Kronfeld-Schor et al., 2013; Helfrich-Förster et al., 2021; Wehr and Helfrich-Förster, 2021).

In January 2001, I started a temporary professorship for zoology at the University of Regensburg, and in October 2009, I moved to the University of Würzburg to succeed Martin Heisenberg on the chair of Neurobiology and Genetics, which I hold until today. From that time onward, I was established in the German scientific system and from now on I can focus my attention on science. A timeline of my scientific discoveries and key life events is shown in Figure 3.

## The Larval Clock of *Drosophila Melanogaster*

**Figure 4.**
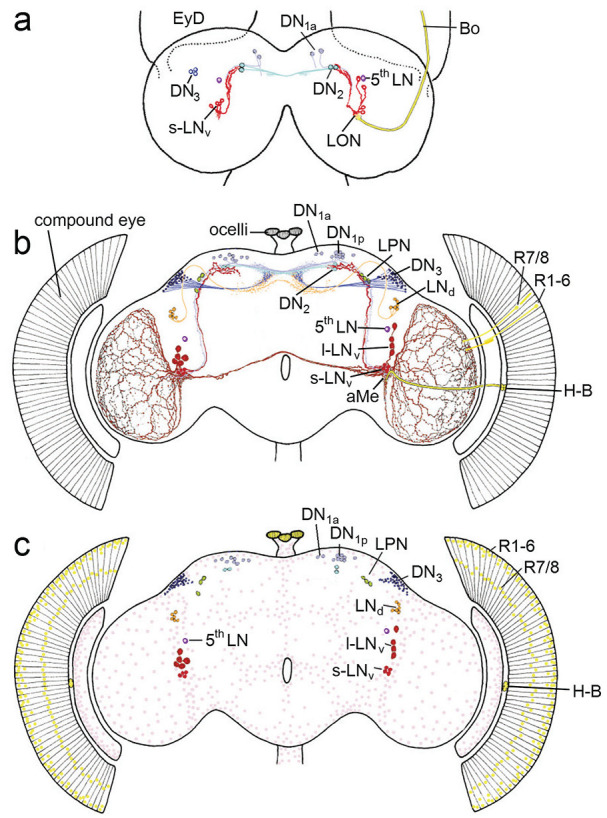
View of circadian clock cells in the larval and adult brain from the year 2000. (a) In the larval brain, the PDF-positive small ventrolateral neurons (s-LN_v_ in red), the fifth lateral neuron (5^th^ LN in lilac), the dorsal neurons 1a (DN_1a_), the dorsal neurons 2 (DN_2_) and few dorsal neurons 3 (DN_3_) were present. The s-LN_v_ have putative dendritic fibers in the larval optic neuropil (LON), where the terminals of the larval photoreceptor nerve, Bolwig’s nerve (BO) ends, and they project to the DN_2_ in the superior protocerebrum. The DN_2_ run to the contralateral dorsal brain hemisphere, while the arborizations of the other clock neurons were still unknown. EyD eye disk from which the compound develops during metamorphosis. (b) Clock neurons of the adult brain. The LON has developed into the accessory medulla (aMe) and the larval photoreceptors into the Hofbauer-Buchner eyelets (H-B). The s-LN_v_ have now dendritic fibers in the aMe and otherwise maintain their projections to the dorsal (superior) protocerebrum. The 5^th^ LN, the DN_1a_ and DN_2_ appear also unchanged, while the l-LN_v_, LN_d_, DN_1p_ and LPN have appeared during metamorphosis and the number of DN_3_ has greatly increased. The PDF positive l-LN_v_ form wide networks in the distal medullae of the optic lobes and connect the aMe of both hemispheres. (c) Schematic representation of all *period* and *timeless* positive cells in the brain and eyes. In addition to the already described clock neurons, the clock is ticking in numerous glia cells that are distributed throughout the surface of the brain and optic lobes (pink). Furthermore, the clock genes are expressed in the nuclei of the photoreceptor cells in the eyes (outer photoreceptor cells R1-6 and inner photoreceptor cells R7/R8) and the ocelli (yellow). Modified after Helfrich-Förster (2003).

The first collaborative paper of Jeff’s group and myself was about the fruit fly larval clock (Kaneko et al., 1997). In this paper, we showed that, in *Drosophila* larvae, the clock genes *timeless* and *period* were cyclically expressed in a subset of the adult clock neurons, and we discovered a fifth lateral clock neuron that previously escaped my attention and that was already functional in the larval brain. We called it the 5^th^ LN (Figure 4; Kaneko et al., 1997), but later it was renamed into the 5^th^ s-LN_v_. In the meantime, we know that the latter name is misleading because the 5^th^ LN has a much larger soma than the 4 PDF-positive s-LN_v_ and that its morphology resembles much more the dorsal lateral neurons (LN_d_) than the ventral lateral neurons (LN_v_) (Schubert et al., 2018). Therefore, I will stick to its original name. Besides the 4 s-LN_v_ and the 5^th^ LN, 2 Dorsal Neurons 1 (DN_1_, later specified as anterior dorsal neurons 1, DN_1a_, Shafer et al., 2006), the 2 Dorsal Neurons 2 (DN_2_), and faintly very few DN_3_ were stainable in the larval brain (Figure 4a). All other clock neurons appeared to be undeveloped. In this study, we also discovered for the first time the close vicinity of the s-LN_v_ and their putative dendrites to the terminals of the larval photoreceptor nerve, also called Bolwig’s nerve (Figure 4a). Furthermore, Maki Kaneko detected 3 new clock neurons by anti-TIM staining in the posterior lateral brain of adult flies. She published this finding together with many more details in an elaborate paper with Jeff (Kaneko and Hall, 2000). These 3 neurons were later called Lateral Posterior Neurons (LPN) and characterized in detail only in 2022 (Reinhard et al., 2022a).

In parallel to the development of *period* and *timeless* positive neurons in the larval brain, I studied the development of the PDF-positive clock neurons (which I then called PDF medulla neurons, PDFMe) more closely and found that, in contrast to the s-LN_v_, which are present from the first larval instar onward and remain unchanged throughout development, the l-LN_v_ (medulla tangentials) are only detectable in the middle of pupal development and their neurites appear fully developed immediately before the adults hatch from the pupae (Helfrich-Förster, 1997). I also described for the first time few weakly stained PDF-positive neurons in the superior brain, which I called PDFCa neurons, PDF-positive neurons around the esophageal foramen (PDFTri neurons), and PDF-positive neurons in the abdominal ganglion (not shown). All these cells do not express the clock genes and are consequently no clock-neurons. The PDFCa neurons are most probably corazonin-containing neurosecretory cells of the *pars lateralis*, which are strongly stained by anti-PDF in flies with strong photoperiodic responses and might be involved in seasonal adaptation (Hamanaka et al., 2007; Manoli et al., 2023). The PDFTri neurons are only present around eclosion and might be involved in its regulation (Selcho et al., 2018).

## The Pdf-Positive Clock Neurons Appear Necessary and Sufficient for Adult Locomotor Activity Rhythms

To prove whether the PDF-positive s-LN_v_ and l-LN_v_ are indeed required for rhythmic behavior, I took advantage of the *disco*^*1*^ mutants mentioned above after finding that individual *disco*^*1*^ flies occasionally possess few PDF neurons. I decided to test whether the presence of these neurons leads to rhythmic behavior by recording the locomotor activity of 357 mutant flies and then immunostaining their brains with anti-PDH. I found that 4 flies still possessed 1 to 2 PDF neurons that sent their projections to the superior lateral protocerebrum, and all 4 flies showed free-running circadian rhythms (Helfrich-Förster, 1998; reviewed in Helfrich-Förster, 2014). This strongly suggests that PDF neurons are necessary and sufficient for adult locomotor rhythms, and that the superior protocerebrum is an output area, in which circadian signals are transferred to downstream neurons. In fact, the superior protocerebrum contains the insect hormonal centers (the neurosecretory cells of the *pars intercerebralis* and *lateralis*, Figure 2), which are most likely homologous to the neurosecretory cells in the paraventricular hypothalamus that project to the pituitary.

In collaboration with Jeff Hall’s group, we also found that PDF levels in the s-LN_v_ terminals in the superior protocerebrum oscillate in a circadian manner, emphasizing the importance of these terminals for circadian rhythmicity (Park et al., 2000). Furthermore, in collaboration with the group of Stephan Schneuwly, we have found that ectopic expression of the *Pdf* gene in non-clock neurons projecting to the superior protocerebrum and apparently releasing PDF into this brain region in a non-circadian manner leads to strongly disturbed circadian rhythms (Helfrich-Förster et al., 2000). The parallel findings of Paul Taghert’s group that a *Pdf* null mutant (*Pdf*^*0*^) shows severely disturbed activity rhythms (Renn et al., 1999) demonstrates the involvement of the neuropeptide PDF and the clock neurons, in which it is expressed, in circadian behavior.

## The Accessory Medulla

Both types of PDF neurons arborize in the accessory medullae (aMe), small neuropils at the base of the medullae (Figures 1d and 4b), which are in the meantime regarded as the circadian pacemaker center of probably all insects, a fact that was nicely demonstrated in cockroaches by transplantation experiments, electrophysiological recordings and Ca^2^ ^+^-imaging (Reischig and Stengl, 2003; Wei et al., 2014, and other work of the Stengl group). The aMe of cockroaches are rich of neuropeptides as are the SCN of mammals, and both, the aMe and the SCN are innervated by photoreceptor cells (Helfrich-Förster, 2004; Mieda, 2020; see Figure 4b for *D. melanogaster*). This strongly suggests that the aMe of insects and the SCN of mammals are analogous structures that house the master clocks of the brain. In *D. melanogaster*, PDF was the first neuropeptide found in clock neurons and the aMe (Figure 4b). However, in the meantime, we and others identified many more neuropeptides that are used as neuromessengers by the clock neurons, and we know that many clock neurons arborize in the aMe (see below). Like cockroaches and mammals, some fruit fly clock neurons (e.g., the LPN) possess up to 4 different neuropeptides with putative different functions (Reinhard et al., 2024). For non-neurobiologists, it should be noted that neuropeptides are ideal clock messengers because they modulate the activity of other neurons in a slow and long-lasting manner instead of triggering fast and short-lasting action potentials.

The aMe gets indirect light input from the compound eyes in perhaps all insects (el Jundi et al., 2011; Li et al., 2018; Arnold et al., 2020; reviewed in Helfrich-Förster, 2019). In addition, it seems to be directly innervated by extraretinal photoreceptors. In holometabolic insects such as flies, the aMe stem from the larval optic neuropils that are innervated by larval photoreceptors or stemmata (Figure 4a; Hagberg, 1986). In *D. melanogaster*, 8 of 12 larval photoreceptors per hemisphere degrade during metamorphosis but 4 survive and are transformed into the Hofbauer-Buchner eyelets that move from the anterior part of the larvae to the posterior margin of the adult compound eye, change their photopigment and develop light-shielding pigment granula (Hofbauer and Buchner, 1989; Malpel et al., 2002; Yasuyama and Meinertzhagen, 1999; Helfrich-Förster et al., 2002, 2007). This transformation alone indicates an important role in the adult insect. Nevertheless, it took us more than 20 years to find out that the Hofbauer-Buchner eyelets mediate high-intensity light adaptation of *D. melanogaster*’s clock (Schlichting et al., 2019b).

## *Drosophila’s* clock is entrained by retinal and extraretinal photoreceptors

As explained above, extraretinal photoreceptors play a prominent role in the entrainment of circadian clocks to environmental light-dark cycles (reviewed in Helfrich-Förster, 2019). In the case of *D. melanogaster*, this became already clear during my diploma work, because complete eye- and ocelli-less *so*^*1*^ and *sol*^*1*^; *so*^*1*^ mutants could nicely entrain to light-dark cycles (Figure 1c). From that point on, I was curious to identify these extraretinal photoreceptors. The Hofbauer-Buchner eyelets were my first guess, because they were still present in eyeless *so*^*1*^ (Hofbauer and Buchner, 1989) and their terminals nicely overlap with putative dendrites from the PDF-positive s-LN_v_ and l-LN_v_ (Figure 3b) (Schlichting et al., 2014, 2016); However, the Hofbauer-Buchner eyelets could not be the sole extraretinal photoreceptors because also flies without them and without functional compound eyes and ocelli were able to entrain (Helfrich-Förster et al., 2001).

### Cryptochrome

Again, Jeff Hall was very helpful in solving the photoreceptor problem, since he introduced me to Ralf Stanewsky, who was postdoc in his lab in the 90ties. Ralf was interested in identifying new clock components and had developed a screen, in which he chemically mutagenized transgenic flies expressing a Period (PER)-luciferase fusion protein as an *in vivo* reporter for PER. This enabled him to identify mutants with disturbed PER oscillations in peripheral tissues in living flies. Among the first mutants he found was the *cryptochrome*^*baby*^ (*cry*^*b*^) mutant, that carried a point mutation in the flavin-binding domain of the blue-light photopigment Cryptochrome (CRY) (Stanewsky et al., 1998). *cry*^*b*^ mutants lacked phase-shifting responses to brief light pulses, and *cry*^*b*^ mutants with additionally impaired photosensitivity in the compound eyes showed only weak entrainment to light-dark cycles. Since I was able to record fly locomotor activity for up to 1 month, we started to collaborate and tested the *cry*^*b*^ mutants in different genetic backgrounds and light programs as well as the effects of *cry* overexpression in the circadian clock neurons. In collaboration with Ralf, Patrick Emery and Michael Rosbash, we found that CRY is a deep brain photoreceptor that mediates entrainment and is most likely working in the clock neurons themselves (Emery et al., 2000b). Later, we found in parallel to the group of Paul Hardin that CRY is indeed expressed in about half of the clock neurons, in the compound eyes and in few other brain cells (Yoshii et al., 2008; Benito et al., 2008). Most interestingly, in the compound eyes and other peripheral oscillators, CRY seems to be in involved in the core molecular clock, meaning that its absence provokes arrhythmicity and that is how it was detected (Stanewsky et al., 1998). Most other animals have a different form of CRY, also called mammalian CRY (see Deppisch et al., 2022, 2023 for an overview). Mammalian CRY is light-insensitive and part of the core molecular clock even in the circadian pacemaker centers of the brain. Thus, CRYs are versatile molecules that may fulfill different roles. In *D. melanogaster* (and birds), CRY appears to work additionally as magnetoreceptor, although there is some debate (Gegear et al., 2008; Yoshii et al., 2009a; Fedele et al., 2014; see also Bassetto et al., 2023; Kyriacou, 2024; Reppert, 2024 for a debate), and there is some evidence that it influences photoreception in the eyes by interacting with the phototransduction cascade (Mazotta et al., 2013; Schlichting et al., 2018). Probably not all CRY functions have been clarified yet.

### Rhodopsin 7

CRY was immediately propagated as the circadian photoreceptor of fruit flies, but to me it was always clear that CRY is just one of the many fly photopigments that entrain the circadian clock to light-dark cycles. Together with Ralf, Jeff and Alois Hofbauer, we showed that one must eliminate all fly photoreceptors (CRY, the compound eyes, the ocelli and the Hofbauer-Buchner eyelets) to lose entrainment to light-dark cycles (Helfrich-Förster et al., 2001). *glass*^*60j*^ *cry*^*b*^ double mutants are flies that lack all known photoreceptors and, indeed, they cannot anymore entrain to light-dark cycles. Nevertheless, these mutants respond with an increase in activity to lights-off (Helfrich-Förster et al., 2001) meaning that they possess at least one additional photopigment that mediates this response. A putative candidate for this photopigment is the enigmatic Rhodopsin 7 (Rh7) which I started working on in 2000 after it was detected in the genome (Adams et al., 2000). Rh7 turned out to be highly conserved among the *Drosophila* genus and present in most arthropods (Senthilan and Helfrich-Förster, 2016). Nevertheless, our and the work of others on Rh7 was of limited success so far, meaning that we still don’t know exactly how Rh7 works (Grebler et al., 2017; Kistenpfennig et al., 2017; Ni et al., 2017; reviewed in Senthilan et al., 2019). Most likely, Rh7 has a critical role in the adaptation of behavior to light, most likely in avoiding harmful light as was shown for Rh7 in multidendritic neurons of *D. melanogaster* (Lazopulo et al., 2019). Consistent with this view, the groups of Denise and Craig Montell showed most recently that Rh7 outside the eyes drives an aversion to blue light (Meyerhof et al., 2024). Future studies must reveal the precise function and location of Rh7. Rh7 appears to be expressed in some clock neurons, but this seems not to be its main expression site (Senthilan et al., 2019).

### The Interplay Between CRY and Rhodopsins

But now back to the “conventional” 6 Rhodopsins and CRY. There is plenty of evidence that they fulfill non-redundant roles in circadian photoreception (Rieger et al., 2003; Yoshii et al., 2015), which is understandable because they work via different mechanisms. Light-activated CRY causes the degradation of the clock protein timeless (TIM) in the clock neurons, which leads to an immediate phase shift of the molecular clock and, under constant light, to the permanent degradation of TIM, thereby stopping the clock and making the flies arrhythmic (Ceriani et al., 1999; Busza et al., 2004; Rosato et al., 2001). Therefore, *cry*^*b*^ and *cry*^*0*^ mutants show only small phase shifts to light pulses (Kistenpfennig et al., 2012), need several days to re-entrain their activity rhythms to a phase-shifted light regime (Emery et al., 2000b) and remain rhythmic under constant light, even at high intensities (Emery et al., 2000a; Helfrich-Förster et al., 2001; Yoshii et al., 2004; Rieger et al., 2006). In contrast, the photoreceptors of the H-B eyelets and compound eyes signal via neurotransmitters (mainly acetylcholine and histamine) to the clock neurons (Schlichting et al., 2016, 2019c; Alejevski et al., 2019; Xiao et al., 2023). They seem to have different roles in entrainment (Schlichting et al., 2014, 2015a, 2016) and most of these photoreceptor inputs are indirect, meaning that they work via various interneurons (Reinhard et al., 2024). The molecular mechanisms of how these light signals are transferred to the molecular clock machinery are still largely unknown. For sure this transfer is indirect and slower than the action of CRY, and constant light via the eyes does not provoke arrhythmic behavior by stopping the clock. Nevertheless, constant light via the eyes leads to prominent changes in period length (the so-called parametric effects of light) (Yoshii et al., 2004; Rieger et al., 2006). Most interestingly, constant light sensed via the eyes changes the clock speed differently in different clock neurons, meaning that the molecular clock of some neurons is accelerated and in other neurons it is slowed down (Rieger et al., 2006; see below).

In several aspects, the effects of CRY, the HB eyelets and the eyes on entrainment are antagonistic to each other (Schlichting et al., 2016). Clear examples of this antagonism are the entrainment to long summer photoperiods and the entrainment to light-moonlight cycles. Under long photoperiods, wild-type flies try to track dawn and dusk with their morning and evening activities, respectively (Rieger et al., 2003). Eyeless *so*^*1*^ or *cli*^*eya*^ mutants are unable to do so, meaning that under long photoperiods their morning activity, if present at all, occurs after dawn and their well pronounced evening activity occurs clearly before dusk. In other words, the morning and evening activity peaks of eyeless flies come close together, because the phase-advancing effects of the eyes on morning activity and the phase-delaying effects on evening activity are lacking. Consequently, eyeless flies cannot track dawn and dusk with their activity, while flies without CRY can still do so perfectly. *cry*^*0*^ and *cry*^*b*^ mutants can even “better” track dawn and dusk than wild-type flies, meaning that they can track dawn and dusk even under very long days, under which wild-type flies cannot anymore follow (Rieger et al., 2003; Kistenpfennig et al., 2018). This indicates CRY has an opposite effect on the phases of morning and evening activities than the eyes: it delays morning activity and advances evening activity (Kistenpfennig et al., 2018). At the molecular level, CRY signaling dampens the amplitude of PAR-domain protein 1 (PDP1) oscillations in most clock neurons during long days, whereas signaling from the visual system increases these amplitudes (Kistenpfennig et al., 2018). Thus, light inputs from the 2 major circadian photoreceptors, CRY and the visual system, have opposite effects on day length adaptation. Their tug-of-war appears to determine the precise phase adjustment of evening activity (Kistenpfennig et al., 2018). A similar effect was observed under moonlit nights. When wild-type flies are exposed to artificial moonlight, they shift large parts of their evening activity into the early night, while eyeless flies do not show any shift (Bachleitner et al., 2007) and *cry*^*0*^ mutants shift evening activity to a much greater extent into the night than wild-type flies (Zurl et al., 2022). Again, the compound eyes are responsible for delaying evening activity into the night, while CRY keeps it in the day. The balance of the 2 effects appears essential for a wild-type phase of activity.

### The Role of the Compound Eyes for Direct Light Effects (Masking)

The compound eyes have another important role in the control of activity. Light perceived via the eyes, allows the flies to follow gradual changes in light intensity precisely with their activity (Rieger et al., 2007; Schlichting et al., 2015a) and this works even in the absence of a functional clock (Schlichting et al., 2015b). In other words, under simulated natural dawn and dusk, the activity of arhythmic *per*^*0*^ mutants is barely distinguishable from that of wild-type flies; however, this is not the case if *per*^*0*^ mutants possess CRY but lack the eyes (Schlichting et al., 2015b). This indicates that the eyes allow clock mutants to exhibit almost normal activity patterns under natural conditions and can explain results gained with different clock mutants in nature (Daan et al., 2011; Vanin et al., 2012). In nature, the cyclic changes in temperature contribute to synchronization (Yoshii et al., 2009b), and natural-like temperature cycles can provoke almost normal activity rhythms even in clock-less flies (Bywalez et al., 2012).

Nevertheless, this does not mean that the circadian clock has no selective advantage. In *D. melanogaster*, a functional clock appears to suppress futile activity in the heat of the midday and during the darkness of the night (Menegazzi et al., 2012; Schlichting et al., 2015b). Furthermore, a functional clock is important for a normal lipid metabolism and resistance to starvation (Schäbler et al., 2020; Amatobi et al., 2023) and for survival in competition with wild-type flies (Horn et al., 2019). The role of the eyes in accurately tracking environmental light changes can be seen as a direct effect of light on activity, also known as masking, as it is independent of the circadian clock and masks its output.

## The Dual Oscillator System: Morning And Evening Clocks

**Figure 5.**
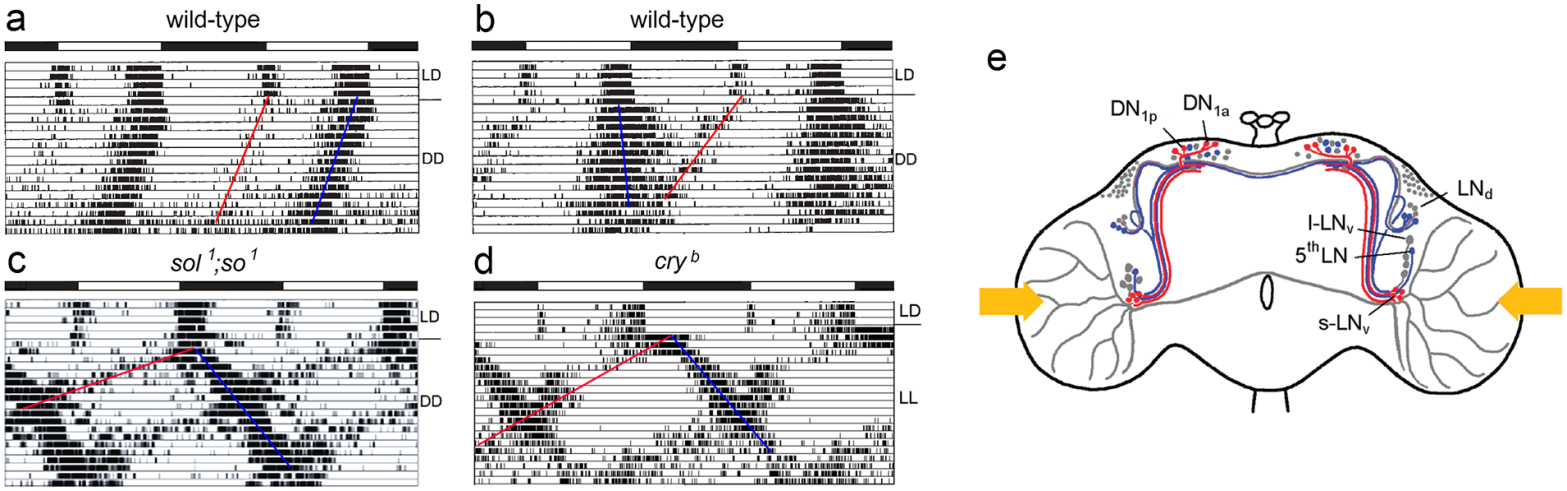
(a, b) Double-plotted actograms of wild-type flies in which the morning activity (red line) persists in constant darkness (DD). In **a**, it free-runs in parallel to the evening activity (blue line), while it runs with a shorter period than the evening activity in **b**. (c) Typical activity pattern of a *sol*^*1*^; *so*^*1*^ mutant that reveals 2 free-running activity components under DD. Both appear to start from evening activity, but it is well possible that the morning component (red line) is the expression of the morning oscillator that free-runs invisibly during the first 2 days in DD. (d) Typical activity pattern of a *cry*^*b*^ mutant in LD and constant light (LL) that largely resembles the activity pattern of the *sol*^*1*^; *so*^*1*^ mutant. (e) Scheme showing morning (red) and evening (blue) clock neurons in the *Drosophila* brain. The yellow arrows indicate the light-input to the l-LN_v_. Labeling of clock neurons as in Figure 4. Modified from Helfrich-Förster (2014).

With their bimodal activity pattern, composed of morning and evening activity that are separated by a siesta (Figure 1c), fruit flies are not alone. Bimodal activity is present in many animals, including birds and mammals (Aschoff, 1966). In 1976, Pittendrigh and Daan observed in nocturnal rodents that morning and evening activity respond differently to light. The period length of morning activity is shortened by light, while the one of evening activity is lengthened. These different responses to light provoke the above-mentioned coupling of morning activity to dawn and of evening activity to dusk under increasing photoperiods that is evident in different species, including fruit flies. Pittendrigh and Daan (1976) assumed that 2 separate circadian oscillators with different sensitivity to light drive morning and evening activity. This model accounts for many observed circadian activity patterns including internal desynchronization into 2 free-running rhythmic activity components that I have observed in the eyeless *Drosophila* mutants with tiny optic lobes (Figure 1c) and rarely in wild-type flies (Figure 5b; Helfrich-Förster, 2000). Usually, the morning activity diminishes when flies are transferred to constant darkness (DD), but sometimes it remains clearly visible and free-runs in parallel to the evening activity (Figure 5a). The interesting cases are the ones in which morning and evening activity do not run in parallel (Fig. 5b). Although these cases are rare, they clearly indicate that independent morning and evening oscillators exist in the fly brain that are usually coupled with each other but can desynchronize under certain circumstances (Helfrich-Förster, 2000, 2001).

In the eyeless *Drosophila* mutants with tiny optic lobes morning and evening activity bouts are far apart under light-dark cycles since morning activity starts already before lights-on and evening activity after lights-off resulting in a long siesta and shorter night sleep in comparison to wild-type flies (compare Figure 5c with Figure 5a). At the first glance, this appears illogical because the eyes advance morning and delay evening activity, and these mutants have no eyes; but there are other reasons, as will be explained below. An early onset of activity indicates a short-period clock, while late activity is usually caused by long-period clocks. Indeed, the late evening activity of the mutants free-runs with a long period after transfer into DD (Figure 5c). The morning activity initially disappears after the transition to DD, but a short-period component splits from the evening activity bout, possibly originating from the morning oscillator, which runs invisibly with a short period during the first days of DD (Figure 5c). Most interestingly, we found an almost identical activity pattern in *cry*^*b*^ (and *cry*^*0*^) mutants after these were transferred to constant light (LL) (Figure 5d). In *cry* mutants solely the compound eyes are responsible for the parametric light effects on morning and evening oscillators (period shortening of the morning oscillator and period lengthening of the evening oscillator) without the disruptive effect of CRY that would make the flies arrhythmic in LL (Rieger et al., 2006). Consequently, we could see the differential effects of light on the free-run of morning and evening oscillators that were predicted by Pittendrigh and Daan (1976) (Figure 5d).

The anatomical basis of these morning and evening oscillators remained elusive until Grima et al. (2004) and Stoleru et al. (2004) genetically manipulated the clock neurons. Grima et al. (2004) expressed the *period* gene in different subgroups of clock neurons in a *per*^*0*^ mutant background, whereas Stoleru et al. (2004) ablated subgroups of the clock neurons by expressing a cell death gene selectively in them. Both laboratories found that— dependent on the manipulation—either the morning or the evening activity bout disappeared. Consequently, they concluded that the PDF-positive s-LN_v_ must control the morning activity bout and the LN_d_ control the evening activity bout. Certainly, this initial morning and evening oscillator model was much too simple, but it set the stage for further investigations. The reader is referred to Helfrich-Förster (2009), Yoshii et al. (2012) and Helfrich-Förster (2014) for a more detailed view on the dual oscillator model.

For me it was important to know, which clock neurons drive the free-running short and long period rhythms in eyeless mutants with tiny optic lobes under DD and of *cry*^*b*^ mutants under LL conditions. To find this out, we immunostained fly brains with anti-PER and anti-TIM on that day in DD or LL, respectively, at which the 2 free-running components were completely out of phase (Rieger et al., 2006; Yoshii et al., 2009c). As presumed, we found that the s-LN_v_ control the component free-running with short period (corresponding to the morning oscillators) (red in Figure 5e), while the 5^th^ LN and about half of the LN_d_ control the component free-running with long period (corresponding to the evening oscillators) (blue in Figure 5e) (Rieger et al., 2006; Yoshii et al., 2009c). Furthermore, we found that about half of the dorsal neurons (DN_1p_) are morning and the other evening oscillators (red and blue in Figure 5e). Thus, the LN_d_ and DN seemed to consist of morning and evening oscillators and to be heterogenous groups of clock neurons. At that time, we did not have the tool to classify these groups in more detail. Furthermore, we completely disregarded the DN_3_ in our analysis since these are difficult to quantify due to their small cell body size.

The next open question to be addressed was how tiny optic lobes associated with altered morphology of the PDF-positive l-LN_v_ could cause the same dissociation of morning and evening activities under DD as the absence of functional CRY in LL. The answer lies in the function of the l-LN_v_ and the neuropeptide PDF released from them. The l-LN_v_ respond to light with a depolarization, an increase in Ca^2+^ levels, and putatively with a release of PDF during the day (Shang et al., 2008; Sheeba et al., 2008; Fogle et al., 2011; Liang et al., 2016). PDF released from the l-LN_v_ into the aMe acts on all the PDF receptor-expressing clock neurons that have neurites there, and these include the morning and evening neurons (Im and Taghert, 2010). In the evening neurons, PDF leads to a clear phase delay (Yao and Shafer, 2014; Liang et al., 2016, 2017; Menegazzi et al., 2017; Schlichting et al., 2019c; Vaze and Helfrich-Förster, 2021), which would result in a period lengthening under LL. Whether PDF leads to the expected phase advances (period shortening) in the morning neurons is less clear, but what is known is that a depolarization of the morning neurons only causes advances and no delays (Eck et al., 2016). This indicates that excited M neurons are potent accelerators of the clock. In summary, increased PDF release from the l-LN_v_ under LL may speed up and slow down the clock in the morning and evening neurons of *cry* mutants, respectively. In the mutants with tiny optic lobes, the l-LN_v_ form a dense PDF fiber network in the dorsal protocerebrum instead of the medulla (Figure 1d), and PDF signaling to morning and evening neurons, both of which branch in the dorsal brain, may already be increased in DD, leading to internal desynchronization of morning and evening activity. Indeed, we could demonstrate a positive correlation between the number of PDF fibers in the dorsal brain and the degree of internal desynchronization between morning and evening activity (Wülbeck et al., 2008). Furthermore, internal desynchronization disappeared in the absence of PDF showing that PDF and not the altered brain morphology of the mutants is the cause of the accelerated morning and decelerated evening oscillators (Wülbeck et al., 2008). A more detailed description of the role of PDF in the dual-oscillator model of rhythm control can be found in Helfrich-Förster (2014).

The power of the dual oscillator model is that it explains observed adaptations to seasonal changes in day length. That PDF is crucial for these adaptations was shown by my group in several studies. Most importantly, the density of PDF fibers in the dorsal protocerebrum was significantly higher in *Drosophila* species of the *virilis* group, which stemmed from high latitudes and were exposed to extremely long photoperiods in the summer as compared to species stemming from lower latitudes (Hermann et al., 2013; Menegazzi et al., 2017; Beauchamp et al., 2018; Helfrich-Förster et al., 2020; Manoli et al., 2023). This is associated with an improved ability to delay evening activity until dusk during long photoperiods and could be of adaptive significance in these species.

## The Clock Network of *D. Melanogaster—*Refined Versions

**Figure 6.**
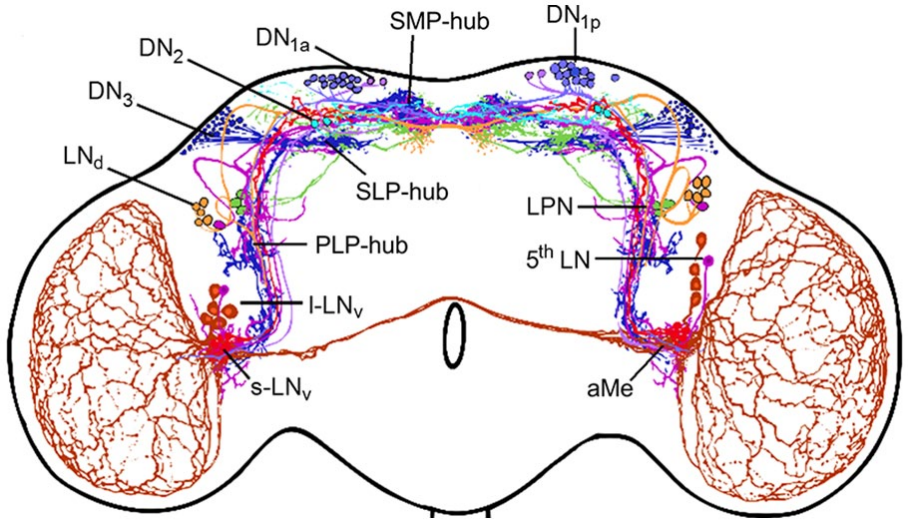
View of circadian clock cells and their branches in the adult brain from the year 2022. This reconstruction comes from antibody staining and driver lines. New fiber hubs became visible in the clock network, one in the superior median protocerebrum (SMP-hub), another in the superior lateral protocerebrum (SLP-hub) and the third one in the posterior lateral protocerebrum (PLP-hub). Otherwise, labeling as in Figure 3. Modified from Reinhard et al. (2022b).

Over the years, the circadian clock network of *D. melanogaster* has been further refined. Immunocytochemical studies revealed that besides PDF many additional neuropeptides are expressed in the different subsets of clock neurons. Among these are Ion Transport Peptide (ITP), Allatostatin A and C, short Neuropeptide F, Diuretic Hormone 31 and 44, Trissin, CCH amide1, CNM amide and Proctolin (Johard et al., 2009; Hermann et al., 2012; Hermann-Luibl et al., 2014; Chen et al., 2016; Fujiwara et al., 2018; Diaz et al., 2019; Ni et al., 2019; Reinhard et al., 2022a, 2024). Antibodies against these neuropeptides together with reporter gene driven clock neuron labeling revealed the neurites of almost all clock neurons (Figure 6). In comparison to Figure 4b, Figure 6 shows dense fiber networks in the superior lateral and medial protocerebrum, close to the neurosecretory centers of the *pars intercerebralis* (PI) and *pars lateralis* (PL), and in the posterior lateral protocerebrum (Reinhard et al., 2022b). This indicates that, besides the aMe, there are additional fiber hubs serving communication between the clock neurons, communication from other neurons to the clock network, and from circadian clock neurons to downstream neurons. An increasing number of available *gal4* or *split-gal4* lines allowed the selective labeling and manipulation of individual clock neurons unraveling their function in increasing detail (Sekiguchi et al., 2020). Furthermore, new techniques such as *transTango* (Talay et al., 2017) allowed the identification of postsynaptic partners of individual clock neurons.

Another era began with the electron microscopic data sets of 2 female *Drosophila* brains, the hemibrain and the Flywire connectome (Scheffer et al., 2020; Dorkenwald et al., 2023), which allowed the annotation of all clock neurons, including their connections to each other and their synaptic inputs and outputs (Shafer et al., 2022; Reinhard et al., 2022b, 2024). Especially the Flywire connectome, which includes the connections between the 2 brain hemispheres and the light input pathways from the compound eyes, allowed the detailed characterization of contralaterally projecting clock neurons and their mostly indirect connections to photoreceptor cells (Reinhard et al., 2024).

**Figure 7.**
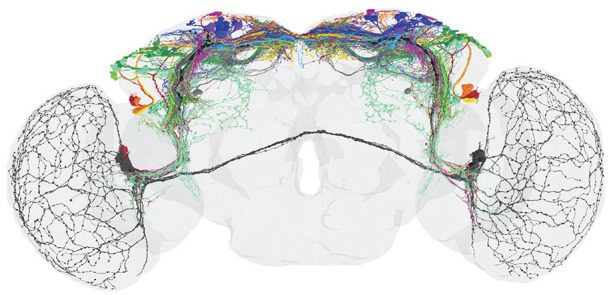
View of circadian clock cells and their branches in the adult brain from 2024. This clock neuron reconstruction comes from electron microscopy (connectomics) backed-up with antibody staining, driver lines and multicolor flip out experiments (Reinhard et al., 2024). The clock cells and brain regions are not highlighted and the colors from Figures 4 and 6 have not been adopted. Note that the number of fibers in the superior median and lateral protocerebrum has increased significantly compared to Figure 6. From Reinhard et al. (2024).

It turned out that the heterogenous group of DN_1p_ consists of at least 5 subgroups and that the DN_3_ contain ~86 clock neurons per brain hemisphere instead of ~40. Thus, the entire clock network consists of ~240 instead of the so far assumed ~150 clock neurons and the network in the superior protocerebrum is even denser as assumed in 2022 (compare Figure 6 with Figure 7).

It is impossible to mention all the new details about the clock neurons and their putative function in the whole circadian system in this review. Here, the reader is referred to the original publications. Let me just summarize the most important finding that emerged from recent anatomical and functional studies. As already indicated by previous results (e. g. Yao and Shafer, 2014), the original hierarchical view of the clock network with the lateral neurons and in particular the PDF neurons as the main circadian pacemakers proved to be too simplistic. Other neurons, in particular the many dorsal clock neurons appear as important as the PDF neurons or can at least compensate for their loss (Delventhal et al., 2019; Schlichting et al., 2019a; Sekiguchi et al., 2024). This suggests that the control of circadian behavior is distributed among several subgroups of clock neurons that interact in a complex network as illustrated in Figure 7. It will be most exciting to reveal the role of the newly classified dorsal clock neurons (DN_1p_ and DN_3_) in the circadian system of the fly. Most importantly, there are multiple connections between the clock neurons and the hormonal centers in the superior protocerebrum that control physiology, metabolism and sleep (e.g., Barber et al., 2021).

## The Clock Network In Other Arthropods

If you look at my scientific background, it becomes clear that I am not only interested in the clock network of *D. melanogaster* but wanted to know how circadian clocks work in the brain in general. Collaborative research grants from the European Union (*EUCLOCK* with Till Roenneberg as speaker, Marie-Curie Training Networks *Insect Time* and *CINCHRON* with Charalambos Kyriacou as speaker), the German Israeli Foundation (collaborative grant between Guy Bloch and myself), and the German Research Foundation DFG (Collaborative Research Center *Insect Timing* with myself as speaker) gave me the opportunity to cooperate with other scientists on this endeavor. Together, we were able to partly characterize the circadian clock network in the brain of the honeybee *Apis mellifera* (Fuchikawa et al., 2017; Beer et al., 2018), the ant *Camponotus floridanus* (Kay et al., 2018), the aphid *Acyrthosiphon pisum* (Colizzi et al., 2021, 2023, 2024), the olive fly *Bactrocera oleae* (Bertolini et al., 2018) and several fruit fly species stemming from different latitudes (Kauranen et al., 2012; Bertolini et al., 2019; Hermann et al., 2013; Menegazzi et al., 2017; Beauchamp et al., 2018; Manoli et al., 2023). Most interestingly, all these species possess clock neurons in the lateral and superior protocerebrum, and the neuropeptide PDF was expressed in subgroups of the clock neurons (see also Beer and Helfrich-Förster, 2020).

Currently, the DFG funded priority program “Antarctic Research with comparative investigations in Arctic ice areas” gives Bettina Meyer (AWI, Bremerhaven) and me the opportunity to extend our studies on Antarctic Krill *Euphausia superba* (see Hüppe et al., 2024 for a first paper). Personally, I am very excited about this project because Antarctic krill takes a key position in the Southern Ocean ecosystem, linking energy from the base of the food web into the highest trophic levels. It is therefore important to know how its daily vertical migration comes about. It is most likely the circadian clock of the krill that controls this, and fortunately, this clock is already partly characterized on the molecular level (Biscontin et al., 2017).

## Comparative Aspects Of Insect And Mammalian Circadian Clock Networks

As mentioned above, the insect accessory medulla can be considered an analog of the mammalian SCN, and several interesting parallels can be drawn between the 2 master circadian clocks. Not only are both rich in neuropeptides, but some neuropeptides even appear to fulfill similar functions. For example, the insect PDF has its counterpart in the mammalian vasoactive intestinal neuropeptide (VIP). Both neuropeptides act on homologous G-protein-coupled receptors, whose activation increases cAMP levels (Harmar et al., 2002; Aton et al., 2005; Helfrich-Förster, 2005; Mertens et al., 2005; Lear et al., 2005; Hyun et al., 2005; Maywood et al., 2006). These receptors are expressed in many clock neurons, and PDF and VIP serve to communicate between the different clock neurons, synchronizing their oscillations. Without PDF and VIP (or their receptors), the clock neurons become asynchronous, resulting in weak and eventually arrhythmic activity rhythms. In both cases, the weak activity rhythms have a short period, suggesting that PDF and VIP mainly have period-lengthening effects. In contrast, knock-out of the neuropeptide Ion Transport Peptide (ITP) in *Drosophila* and knock-out of the clock in arginine-vasopressin (AVP)-expressing clock neurons in mice result in a period lengthening of the activity rhythms, suggesting that these 2 neuropeptides predominantly have period-shortening effects (Hermann-Luibl et al., 2014; Mieda et al., 2015). All this is reminiscent of the dual oscillator model proposed by Pittendrigh and Daan (1976), which predicts morning and evening oscillators with different properties. The fruit fly was the first animal in which morning and evening neurons were described at the anatomical level: the small PDF neurons (s-LN_v_) are morning oscillators, while the ITP neurons (fifth LN and 1 LN_d_) are among the evening oscillators. In mammals, morning and evening oscillators have been found in the rostral (anterior) part of the SCN (Jagota et al., 2000; Hazlerigg et al., 2005; Inagaki et al., 2007). Strikingly, the putative morning neurons (VIP neurons in mice and small PDF neurons in *Drosophila*) are in the ventral parts of the master clocks, whereas the evening neurons (AVP or ITP neurons, respectively) are in the more dorsal part of the master clocks. Furthermore, mammalian and *Drosophila* VIP and PDF neurons appear to receive strong light input from the eyes and make connections with ITP and AVP neurons, respectively. More recently, other putative evening neurons have been discovered in the rostral part of the mouse SCN. These are neurons containing the neuropeptide cholecystokinin (CKK) (Xie et al., 2023), some of which also appear to express AVP and prokineticin, as revealed by RNA sequencing analyses (Wen et al., 2020; Xu et al., 2021; summarized in Mieda, 2023). Nevertheless, and in apparent contrast to flies, there appear to be no defined areas of the SCN that only contain morning or only evening oscillators (Mieda et al., 2015). Rather, morning and evening oscillators form distributed networks in the SCN. On closer inspection, this difference between mice and flies is reduced. In *Drosophila*, too, PDF and ITP neurons are not the only morning and evening oscillators (Rieger et al., 2006; Yoshii et al., 2009c; Yoshii et al., 2012). The numerous dorsal clock neurons also appear to consist of morning and evening oscillators. They are much more heterogenous than originally thought, and the role of many of them in the *Drosophila* circadian system is not yet understood (Reinhard et al., 2024). It is highly likely that flies also have distributed networks of morning and evening oscillators, as is the case in mice.

The organization of the circadian master clock is not the only parallel between flies and mice (or, more generally, between insects and mammals). Both master clocks send signals to the neurohormonal systems, which are in the pituitary and pineal gland of the hypothalamus in mice and in the *pars intercerebralis* and *lateralis* in flies, which are connected to the *corpora cardiaca* and *allata* (see Reinhard et al., 2024 for a comparison). The link between the circadian clock and the neurohormonal system ensures not only the circadian release of hormones, but also the seasonal adaptation of physiology and behavior (see below).

Another interesting parallel between insects and mammals (perhaps more generally between invertebrates and vertebrates) is the location of the master clock near the optical system and the use of several retinal and extraretinal photoreceptors to entrain it to the light-dark cycles of the environment (reviewed by Helfrich-Förster, 2019). In this regard, fruit flies appear to be more complicated than mammals because they have light-sensitive CRY in addition to retinal and extraretinal photoreceptor organs (Deppisch et al., 2022). A phylogenetic analysis revealed that the non-light-sensitive mammalian CRY is most likely the original CRY form, and in particular, those insects that are exposed to high levels of light, additionally (or exclusively) possess the light-sensitive form of CRY (Deppisch et al., 2023). Photoprotection or rapid adaptation to light could be one of the possible reasons for the evolution of light-sensitive CRY. Nevertheless, flies without CRY still have multiple photoreceptors for their circadian clock and can perfectly adapt to the light-dark cycles in their environment (Rieger et al., 2003; Schlichting et al., 2015b). In many ways, their entrainment behavior is more similar to that of mammals than to that of wild-type flies (Kistenpfennig et al., 2012).

While there are differences between the circadian systems of mammals and insects, the similarities undoubtedly outweigh the differences, and research on the clocks of insects and mammals has enriched each other.

## Role Of The Circadian Clock In Photoperiodic Responses

Let me come back to one important role of the circadian clock mentioned in the beginning, the measurement of day length that is essential for seasonal adaptation. As Bünning stated already in 1936, preparing in time for the winter is essential for survival. Organisms that fail to do so will ultimately die, while organisms that fail to reproduce at the right time in spring/summer will have no offspring. Bünning therefore concluded that the possession of a circadian clock that provides a reference time necessary for measuring day length represents a considerable selection advantage. I am delighted that Dirk Rieger and I had the opportunity to guest-edit an issue of the Journal of Comparative Physiology A entitled “A clock for all seasons” (Helfrich-Förster and Rieger, 2024). It is now almost 50 years since Colin S. Pittendrigh and Serge Daan published their influential article “A functional analysis of circadian pacemakers in rodents.” V. Pacemaker structure: a clock for all seasons” in the same journal (Pittendrigh and Daan, 1976). Our special issue comprises ten review articles, 5 original research papers and 3 perspective pieces, which collectively address a diverse set of questions pertaining to daily and seasonal timing in invertebrates (primarily insects), birds, and mammals. As true for behavioral rhythms, the overwintering responses of insects are regulated by neuropeptides, and again the neuropeptide PDF, among others, appears to be critically involved in this regulation (Shiga and Numata, 2009; Nagy et al., 2019; Kotwica-Rolinska et al., 2022; Hasebe et al., 2022; Colizzi et al., 2023; Hidalgo et al., 2023; Helfrich-Förster, 2024b).

## Concluding Remarks

During my scientific career, I have been fortunate to work in a very interesting field and on exciting topics. I also came across highly interesting and versatile molecules, the neuropeptide PDF, CRY and the Rhodopsins, of which Rh7 is the most mysterious. Together with other researchers, I have unraveled the circadian clock network in the brain of *D. melanogaster* and other insects. The principal features of this clock network appear largely conserved in all insects and have great similarities to that of mammals and probably other invertebrates and vertebrates. In particular, there are multiple inputs from the photoreceptors to the neuronal clock network, which is highly peptidergic and sends signals to the endocrine system via multiple output pathways. Thus, the work on the fruit fly has advanced our general knowledge about the organization of circadian clocks in the brain. I am honored having laid groundwork for further generations of scientists to discover more about circadian clock networks and their role in seasonal adaptation and survival of not only insects, but also other animals and humans. I wish for these younger ones to find guidance and support as well as motivation and ideas as much as I ever had the privilege to have.
